# Cerebro-Spinal Meningitis in Canada

**Published:** 1872-04

**Authors:** 


### Correspondence.

*To the Editor of the Buffalo Medical Journal*:

Seeing Cerebro-Spinal Meningitis figuring conspicuously in the March number of the Journal. I send you the report of a case which I saw on Easter Sunday.

The patient A. G. I. was in his usual good health on Friday, on Saturday he complained of headache and sick stomach, when his mother gave him a full dose of Cathartic Pills. Nothing very unusual occurred except that free vomiting accompanied the action of the cathartic.

About 10 A. M. on Sunday he complained of numbness in his legs, and told his mother he saw her double. Before 12 he was totally insensible when a well known Physician living in the same Village, was at once sent for, was promptly on hand, and immediately foretold a fatal result.

About 4.30 P. M. I saw the case and found the following symptoms. The pulse, soft, full and very little quickened. The skin cool, but not dry, the pupils dilated unequally, the left most, the mouth nearly closed, lips slightly parted, and covered with clammy mucus, constant motion and clutching of the left arm and hand to the head, no motion, and apparently no sensation in the lower extremeties. While agreeing with the attending physician in his prognosis, we both hoped that there would be time to do something, as the lad was healthy, vigorous, and only 17 years of age. *But, he died at 2 o'clock A. M. on Monday,* his sickness lasting about 14 hours. Cold to the head and a blister, cups with simple mustard Caltplams, Linaments of red pepper and ammonia with turpentine enemeta, and I believe a couple of doses of Iod. and Bromide of potassa were used, yet the result was as I have told you, death in 14 hours. On my way home next day I came to this conclusion that if I ever saw another such case (and I hope I wont) that I shall put the patient in a hot mustard bath, use the cold douche to the head, and whatever other means I can obtain to excite action to the surface, this case clearly convincing me that if the great nervous centres are not relieved at once, by some artificial means you have not time given to depend on the therapeutical action of drugs.

I am, sir, your obedient servant,

YORK, ONT., April 2nd, 1872. R. H. D.

